# Converting Sewage Water into H_2_ Fuel Gas Using Cu/CuO Nanoporous Photocatalytic Electrodes

**DOI:** 10.3390/ma15041489

**Published:** 2022-02-16

**Authors:** N. M. A. Hadia, Ahmed Adel A. Abdelazeez, Meshal Alzaid, Mohamed Shaban, S. H. Mohamed, Bram Hoex, Ali Hajjiah, Mohamed Rabia

**Affiliations:** 1 Physics Department, College of Science, Jouf University, Sakaka P.O. Box 2014, Saudi Arabia; mmalzaid@ju.edu.sa; 2 Basic Sciences Research Unit, Jouf University, Sakaka P.O. Box 2014, Saudi Arabia; 3 Nanoscale Science, University of North Carolina at Charlotte, Charlotte, NC 28223, USA; aabdelh2@uncc.edu; 4 State Key Lab of Metastable Materials Science and Technology, Yanshan University, Qinhuangdao 066004, China; 5 Nanophotonics and Applications Lab, Physics Department, Faculty of Science, Beni-Suef University, Beni-Suef 62514, Egypt; mssfadel@aucegypt.edu (M.S.); mohamedchem@science.bsu.edu.eg (M.R.); 6 Department of Physics, Faculty of Science, Islamic University of Madinah, Prince Naifbin Abdulaziz, Al Jamiah, Madinah 42351, Saudi Arabia; abo_95@yahoo.com; 7 Physics Department, Faculty of Science, Sohag University, Sohag 82524, Egypt; 8 School of Photovoltaics and Renewable Energy Engineering, University of New South Wales, Sydney, NSW 2052, Australia; b.hoex@unsw.edu.au; 9 Electrical Engineering Department, College of Engineering and Petroleum, Kuwait University, Safat 13113, Kuwait; 10 Nanomaterials Science Research Laboratory, Chemistry Department, Faculty of Science, Beni-Suef University, Beni-Suef 62514, Egypt

**Keywords:** hydrogen generation, sewage water, photocatalyst, water spiting, CuO, nonporous

## Abstract

This work reports on H_2_ fuel generation from sewage water using Cu/CuO nanoporous (NP) electrodes. This is a novel concept for converting contaminated water into H_2_ fuel. The preparation of Cu/CuO NP was achieved using a simple thermal combustion process of Cu metallic foil at 550 °C for 1 h. The Cu/CuO surface consists of island-like structures, with an inter-distance of 100 nm. Each island has a highly porous surface with a pore diameter of about 250 nm. X-ray diffraction (XRD) confirmed the formation of monoclinic Cu/CuO NP material with a crystallite size of 89 nm. The prepared Cu/CuO photoelectrode was applied for H_2_ generation from sewage water achieving an incident to photon conversion efficiency (IPCE) of 14.6%. Further, the effects of light intensity and wavelength on the photoelectrode performance were assessed. The current density (J_ph_) value increased from 2.17 to 4.7 mA·cm^−2^ upon raising the light power density from 50 to 100 mW·cm^−2^. Moreover, the enthalpy (ΔH*) and entropy (ΔS*) values of Cu/CuO electrode were determined as 9.519 KJ mol^−1^ and 180.4 JK^−1^·mol^−1^, respectively. The results obtained in the present study are very promising for solving the problem of energy in far regions by converting sewage water to H_2_ fuel.

## 1. Introduction

Photocatalytic materials represent an important class of components for potential applications in renewable-energy-related fields such as solar cells, optoelectronic, and photocatalytic H_2_ production [1,2,3,4]. On the one hand, the production of H_2_ gas from sewage water is a very promising field of renewable energy. This process provides H_2_ gas fuel for different uses of normal life such as cooking and warming, especially in remote regions inside deserts or rural areas. Moreover, this H_2_ gas is used as a fuel for airplanes and aircrafts, in addition to its normal utilization in industrial factories and companies. On the other hand, this photocatalytic reaction removes contamination (sewage water) through conversion into fuel. In addition, the H_2_ fuel is clean energy; its combustion has no side effects. The dependence of the world on this clean energy reduces the use of fossil fuels with their harmful produced gases such as CO_2_, NO_x_, and SO_x_ [5,6,7].

The photocatalysts applied for H_2_ generation reaction must be semiconducting materials such as metal oxides, sulfides, nitrides, or organic [8,9]. Metal oxides have many benefits that qualified them to be ideal photocatalytic materials for H_2_ production such as low cost, stability, and easy preparation [10,11]. There are some methods for enhancing the photocatalytic activity such as increasing the surface area (nanofibers, nanowires, and nanotubes) [12,13,14]. Another way to enhance the photocatalytic activity is through the use of plasmonic materials [15,16], or materials with high thermal capacity such as Cu metal. These materials are effective for light capture and cause electron localization phenomena extended over the composite semiconductor materials, after which the neighbor materials use these phenomena for the H_2_ production process [17]. Previous studies examined the effect of Cu as a plasmonic material in Cu/ZnO for light capture and enhancement of the photocatalytic properties of ZnO [18].

CuO and Cu_2_O are promising materials for renewable energy applications, owing to their bandgap values of 0.7–1.6 eV and 2.0–2.2 eV for CuO and Cu_2_O, respectively [19,20]. These low bandgap values enable these materials to absorb most of the sunlight, which is preferable to large bandgap semiconductors that can absorb 10% to 20% of sunlight [21]. Moreover, the lower bandgap and high absorption efficiency of CuO favor its application (compared with Cu_2_O) in the renewable energy field [22].

In 2014, Li et al. managed to synthesize a nanoporous CuO layer onto Cu foil through the annealing of Cu (OH)_2_ nanowires at 500 °C under an oxygen flow. A thick Cu_2_O interlayer was also formed; this annealing process formed under high oxygen pressure, which is higher than CuO dissociation pressure, so the CuO layer formed at the outer surface of the structure. The Cu/Cu_2_O/CuO structure is used as an electrode for glucose sensing. Sagadevan et al. prepared CuO nanoparticles via the combustion technique for various annealing temperatures (100 °C and 300 °C), with ascorbic acid used as a capping agent [23]. Ragupathi et al. prepared CuO/g-C_3_N_4_ for a water-splitting reaction, but the produced current density (Jph) and the incident photon to current conversion efficiency (IPCE) were very small [24]. Quyen et al. studied the effect of Cu on TiO_2_ for the photocatalytic water splitting reaction and determined that Cu nanoparticles increased the photocatalytic effect very much and increased the rate of H_2_ generation, which resulted from increasing oxygen vacancies in TiO_2_ and the charge transfer process [24]. Shen et al. prepared graphite carbon nitride and decorated this material with CuO for photocatalyst application, and studied the effect of CuO for increasing the efficiency of the catalytic reaction and H_2_ generation, but the rate of H_2_ generation was small [25].

Most of the previous studies on CuO relied on the use of an additional sacrificing agent such as Na_2_SO_3_, Na_2_S_2_O_3_, HCl, and NaOH [26,27,28]. Moreover, the H_2_ rate production was limited [29,30,31,32]. Various fabrication methods of CuO have been reported such as physical sputtering, atomic layer deposition, spray pyrolysis, radiofrequency sputtering, and so on. However, these techniques have drawbacks of high-cost, long fabrication time, and complex fabrication processes [33,34].

This study provides the H_2_ gas fuel from the contamination (sewage water) without using any additional electrolyte. The catalytic electrode is prepared with a low-cost combustion method without using any complex techniques. The prepared electrode has a high J_ph_ in comparison with the previous literature. The produced efficiency is promising for application of this electrode in H_2_ generation from sewage water in industrial applications.

In this study, Cu/CuO photocathode, prepared by an oxidation/combustion process, was evaluated for H_2_ production from a sewage water-splitting reaction without using any sacrificing agent. The influence of various factors such as light intensity and wavelength, temperature, and on/off chopped current was assessed on the photocathode performance. Finally, IPCE was estimated under different monowavelength light, and the mechanism for the sewage water splitting reaction was examined.

## 2. Materials and Methods

### 2.1. Cu/CuO Nanomaterial Preparation

Prior to sample preparation, the Cu foil was cleaned using water, soap, acetone, and ethanol under ultrasonication for 10 min each. The preparation of Cu/CuO was carried out through oxidation combustion of copper foil (99.9%, thickness 0.3 mm) in Nabertherm box furnace (Nitrex Metal Inc., St-Laurent, QC, Canada) at 550 °C for 1 h. Through this combustion process, the Cu metal is oxidized to generate CuO NP material. Then 1 cm^2^ of the Cu/CuO was used as electrode for hydrogen generation under wastewater splitting reaction through electrochemical measurements.

### 2.2. Characterization

X-ray diffraction pattern (XRD, Malvern Panalytical Ltd., Malvern, UK) analyses were carried out using a Bruker D8 advance diffractometer using Cu Kα radiation (wavelength = 0.15418 nm). Field-emission scanning electron microscopy (FE-SEM, Hitachi, S-4800, Schaumburg, IL, USA) was used to assess the morphology. The energy dispersive X-ray analysis (EDAX, Hitachi, Schaumburg, IL, USA) elemental composition and elemental mapping were examined using the EDAX unit attached to the FE-SEM. The optical properties were examined using a double beam spectrophotometer (Perkin Elmer Lamba 950, Shelton, CT, USA).

### 2.3. Electrochemical Measurements

**Figure 1:**
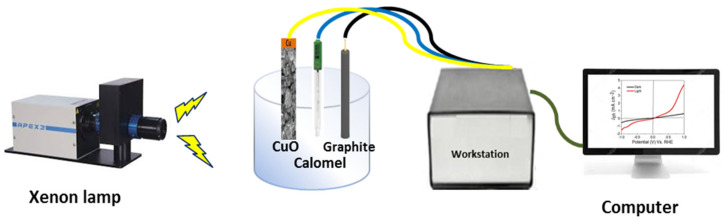
Schematic diagram for photoelectrochemical H_2_ generation process from sewage water.

The H_2_ generation reaction was carried out from sewage water solution (100 mL, pH 5.5) using a three-electrode cell, in which Cu/CuO nanomaterial (1cm^2^), a graphite sheet of the same dimensions, and calomel act as working, counter, and reference electrodes, respectively, as shown in Figure 1. All measurements were carried out using a workstation (CHI660E, Tennison Hill Drive, Austin, TX, USA) in a potential range from −1 to 1 V, a xenon lamp acts as a solar simulator. Some parameters were studied such as the light intensity (25 to 100 mW·cm^−2^), light wavelength (390 to 636 nm), on/off chopped current, and temperature on the electrode performance.

The sewage water was obtained from the drinking water and sanitation of Beni Suef city, Egypt, and the construction was confirmed using gas chromatography–mass spectrometry.

## 3. Results and Discussion

### Morphological, Structural, and Optical Properties

**Figure 2:**
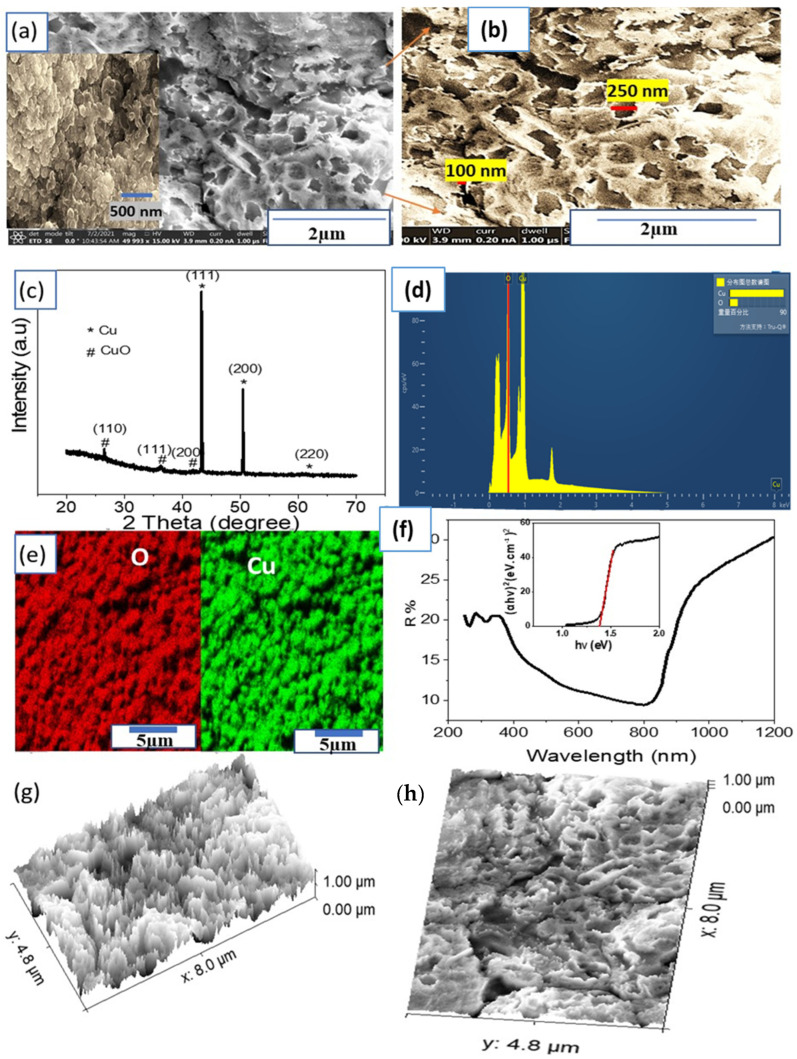
(**a**,**b**) FESEM images, (**c**) XRD pattern, (**d**) EDAX, (**e**) elemental mapping for O (red) and Cu (green), and (**f**) optical reflectance and bandgap (insert) of the CuO NP thin film. (**g**,**h**) The cross section and surface roughness using the modeling program (ImageJ), respectively.

The morphology of the CuO NP, prepared from Cu metal using a simple combustion process in air at 550 °C for 1 h, is displayed in Figure 2a,b. The CuO NP substrate consists of island-like structures with an inter-distance of 100 nm. Each island is highly porous with a pore diameter of about 250 nm. The formation of these pores with high homogeneous distribution on the CuO surface enlarges the surface area. This feature is beneficial for the photocatalytic process through high light absorption efficiency, in which the porous surface acts as a cave for light absorption [7,12].

Figure 2c shows the XRD pattern of the Cu/CuO sample showing the two characteristic peaks of CuO at around 35.6° and 38.5° (JCPDS #41–0254), indicating that CuO NP were formed on the copper foil [35,36,37]. The other three strong diffraction peaks at 43.5°, 50.7°, and 74.5° correspond to the (111), (200), and (220) reflections, respectively, of the face-centered-cubic Cu (JCPDS #02–1225) [36,38]. The crystal size of the CuO nanomaterials is calculated using the Scherrer equation [39,40,41], Equation (1):

(1) D=0.94λ/ßcosθ

where ß is the full width at half maximum (), λ is the X-ray wavelength (CuKα = 0.154 nm), and θ is the Bragg’s angle [42]. From the equation, the average crystal size of CuO is about 89 nm.

The EDAX spectrum (Figure 2d) and EDAX mapping images (Figure 2e) confirm the formation of the CuO NP material where Cu and O peaks are obviously present. The Cu and O elements are homogeneously distributed over the whole examined area.

The optical diffuse reflectance of the CuO NP was determined using a double beam spectrophotometer, as shown in Figure 2f. It is evident that the prepared CuO NP has a high light absorption behavior in a wide optical range (Vis and near IR). This is related to the low reflectance behavior of CuO NP in these regions. The bandgap is calculated using the Kubelka–Munk equation (Equation (2)) [12], as shown in the insert of Figure 2f, where K is the molar absorption coefficient, and S is the scattering factor. From Equation (2), a bandgap of 1.38 eV was determined for the CuO thin film, which is in good agreement with a recent research study [43].

(2) $$K/S=\frac{{\left(1-R\right)}^{2}}{2R}$$

The cross section and roughness is estimated using the modeling program (Image J) as shown in Figure 2g,h. From this figure, the surface roughness appears well with a surface area of 235 µm^2^ in 38 µm^2^.

**Figure 3:**
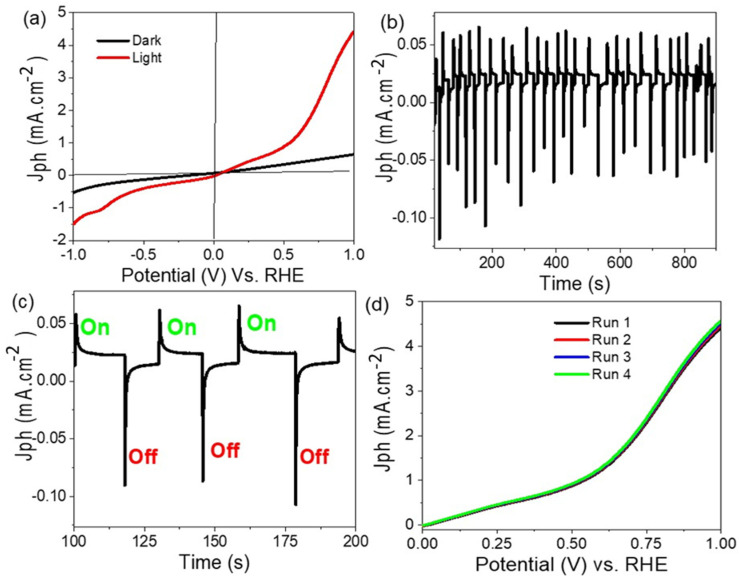
The voltage–current relation for (**a**) Cu/CuO and (**b**,**c**) on/off chopping current (**d**) stability.

The photoelectrochemical (PEC) activity of the Cu/CuO photoelectrode was assessed in sewage water (chemical composition in Table 1) at room temperature (25 °C) with a sweep rate of 1 mV/s under xenon lamp illumination. The PEC measurements were performed in dark and light without optical filters, as shown in Figure 3a. The broad surface area of the prepared nanotextured electrode generates a high density of electron-hole pairs when exposed to light, leading to the splitting of H_2_O molecules to conduct the hydrogen electro-generation reaction. The effect of light on the Cu/CuO photoelectrode generate *J*_ph_ values of −0.07 and 4.7 mA·cm^−2^ at 0 and 1 V, respectively (Figure 3a), although the density of the dark current is very small for the electrode and can be ignored. Therefore, from the *J*_ph_ values, the Cu/CuO with the lowest photogeneration voltage (0.56 V) is a highly efficient electrode for water splitting and H_2_ gas generation.

The substrate Cu metal contributes to a high density of pairs of electrons formed. This will motivate H_2_O molecules to be broken to produce hydrogen when it reaches the CuO surface. The spectral overlap between CuO absorbance oscillator frequencies and the Cu metal oscillator frequency improves the produced photocurrent.

The stability (time-J_ph_ relation) of the prepared Cu/CuO photoelectrode was studied as presented in Figure 3b. At +0.1 V, the produced J_ph_ value under on and off chopped light is mentioned. From the figure, the electrode has high stability and sensitivity to light. From the magnified part in Figure 3c, the change in the J_ph_ values under on and off chopped light appears well. This confirms the high sensitivity of the electrode to light due to the high effect of the light on the electrode. The reproducibility of the electrode for four runs is shown in Figure 3d, in which the voltage–current relation shows the same behavior until four runs. This reproducibility was carried out at 30 °C and in normal light. The standard deviation (SD) value for the Cu/CuO photoelectrode is 0.3%.

**Figure 4:**
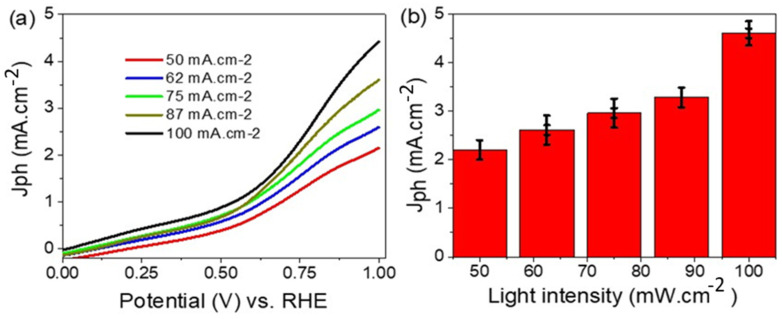
(**a**,**b**) The effect of light intensity on J_ph_ for the Cu/CuO photoelectrode.

The effect of light intensity (50 to 100 mW·cm^−2^) on the Cu/CuO photoelectrode is mentioned in Figure 4a,b. This effect appears clearly, in which the J_ph_ values increase from 2.17 to 4.7 mA·cm^−2^, directly with the light intensity until 100 mW·cm^−2^. This increase is related to the electron–hole pair formation under the increasing light intensity [44], in which many photons activate the active sites on the photocatalytic materials [45]. The J_ph_ represents the electrons collected on the surface of the photoelectrode; this J_ph_ then represented the water splitting and H_2_ generation rate [46,47].

The number of photons (N) is directly proportional to the light intensity (P) as shown in Equation (3). This equation depends on other factors such as wavelength (λ), Planck constant (h), and light velocity (c). From this equation, the N per second is changed from 4 × 10^21^ to 8 × 10^21^ photon/s under light intensity from 50 to 100, mW·cm^−2^, respectively.

(3) N=λP/hc

**Figure 5:**
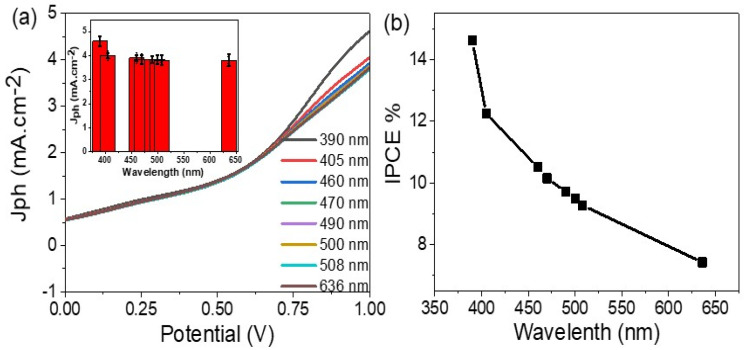
(**a**) The potential J_ph_ relation under different wavelengths (390 to 636 nm) illumination and (**b**) the IPCE for Cu/CuO electrode.

**Figure 6:**
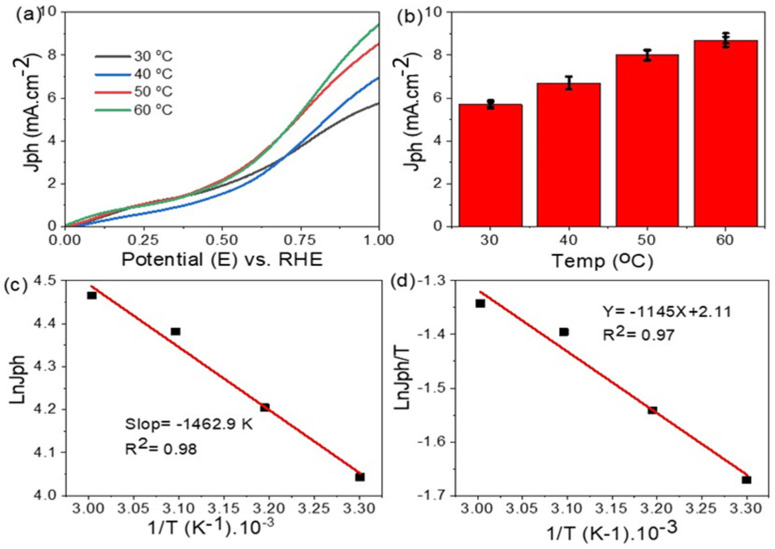
(**a**,**b**) The temperature effect, (**c**) activation energy, and (**d**) heat of reaction for Cu/CuO electrodes.

The effect of the light monowavelength (390 to 636 nm) on the produced J_ph_ for Cu/CuO electrodes is presented in Figure 5a. From this figure, the J_ph_ has varied values under the monochromatic light effect. The optimum J_ph_ value is 4.6 mA·cm^−2^ at 390 nm, in which these values correspond to the optical analyses data (Figure 3). The inset figure in Figure 6a shows this relation clearly.

The incident photon to current conversion efficiency (IPCE) represents the electrons collected on the surface of the photocatalytic materials under the photon flux (Figure 5b). This IPCE value can be calculated from the wavelength values [48], through Equation (4).

(4) $$\mathrm{IPCE}=\frac{J_{\mathrm{ph}}\left(\mathrm{mA}\cdot {\mathrm{cm}}^{-2}\right)\cdot 1240\;\left(V\cdot \mathrm{nm}\right)}{P\left(\mathrm{mW}\cdot {\mathrm{cm}}^{-2}\right)\cdot \lambda \left(\mathrm{nm}\right)}$$

The IPCE is determined at 100 mW·cm^−2^ for the photoelectrode Cu/CuO and presented in Figure 5b. The IPCE values very much depend on monochromatic light, in which the optimum IPCE value is 14.6% at 390 nm. The IPCE values decrease with increasing wavelengths. Therefore, the photocatalytic electrode has the ability for sewage water splitting and H_2_ generation with 14.6% efficiency. This high value was produced without using any additional electrolyte and infers that the electrode converts the sewage water into H_2_ and O_2_ with high efficiency in comparison with other previous literature [29,49,50,51,52,53].

There is an inverse relationship between the number of photons and IPCE. The optimum values occur at low wavelengths, in which the light has a high frequency for transferring most electrons to the conducting band, so the J_ph_ value increases, and thereby the H_2_ production increases [54].

The water splitting reaction for the H_2_ generation process using the Cu/CuO photoelectrode under different temperatures is mentioned in Figure 6a. The J_ph_ values increase from 4.7 to 8.8 mA·cm^−2^ with increasing the temperature from 30 to 60 °C, respectively. This increasing behavior of the J_ph_ indicates the high mobility of the sewage water ions with increasing temperature, in which the high ionic mobility facilitates the H_2_ and O_2_ generation in both sides of the electrochemical cell. So increasing the J_ph_ values with the temperature indicates an increase in the H_2_ generation rate [55,56].

The activation energy (E_a_) for the H_2_ generation can be calculated under different temperatures using the Arrhenius equation (Equation (5)) [57]. This E_a_ depends on the rate of collisions (k is the rate constant) and temperature values in Kelvin (T) using the universal gas constant (R) and Arrhenius constant (A) as the standard constant. From the E_a_ value, the initiator temperature for starting the splitting reaction is determined [58,59,60]. E_a_ values are obtained from slope of ln J_ph_ versus 1/T as shown in Figure 6c. The E_a_ value is 11.8 KJ mol^−1^ for the electrode. The small value of the water splitting reaction indicates the splitting occurs easily for the H_2_ and O_2_ evolution [61].

For calculating the enthalpy (ΔH*) and entropy (ΔS*), Equation (6) is applied (Eyring equation) [62,63]. This equation is similar to the Arrhenius equation, but it contains additional parameters and uses additional constants, in which k_B_ is the Boltzman constant and h is the Planck constant. By applying this equation for the sewage splitting reaction of the Cu/CuO photoelectrode, ΔH* and ΔS* values are obtained as 9.519 kJ mol^−1^ and 180.4 JK^−1^·mol^−1^, respectively (Figure 6c).

Moreover, the H_2_ moles are calculated from the Faraday equation, Equation (7) [64,65], under time change (dt), using the Faraday constant (F). The estimated H_2_ mole for the Cu/CuO photoelectrode is 60 μmol h^−1^ cm^−2^. In addition, a comparison between the present study and the previously reported literature is added in Table 2.

(5) $$k={\mathrm{Ae}}^{-\mathrm{Ea}/\mathrm{RT}}$$

(6) $$k=T\cdot \frac{\mathrm{kB}}{h}\cdot e^{\Delta S/R}\cdot e^{-\Delta H/\mathrm{RT}}$$

(7) $$H_{2}\;\mathrm{mole}=\int_{0}^{t}J_{\mathrm{ph}}\cdot \mathrm{dt}/F$$

**Figure 7:**
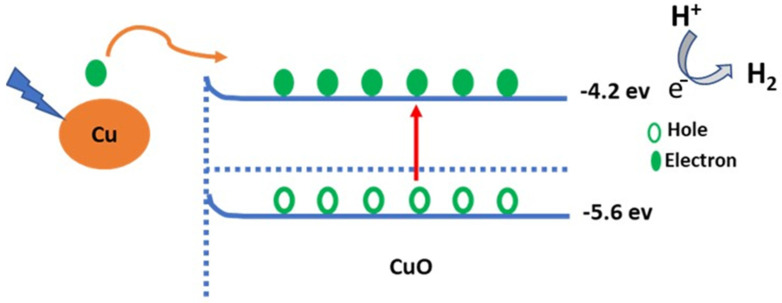
Schematic diagram of the sewage water splitting mechanism.

The mechanism of the CuO photocatalytic materials for the sewage water-splitting reaction is based on the effect of incident light on the motivation of the photoelectrons from the CuO NP material that resulted from the energy band changes (Figure 7). The photoelectrons generated under the effect of light incidence have two steps. First, electron-hole generation, in which the generated electrons leave the holes and transfer to the upper level. The second step is the localized surface plasmonic resonance (LSPR); this resonance process causes the energy transfer [66]. These two steps occurred easily due to the small CuO band gap of 1.39 eV in addition to the absence of depletion regions inside the Cu and CuO nanomaterials. Therefore, the results are the transfer of electrons from the Cu to CuO without any restrictions, and the continuous electrons flow is carried out without any restrictions [67]. These hot electrons cause the generation of J_ph_ under the applied potential [68,69,70]. The experimental image of electrons transfer processes is represented in the optical analyses (Figure 3) and the electrochemical measurements under various effects such as light intensity and light wavelengths. The Cu metal substrate has plasmonic properties that cause the light capture and electron resonance process [52] that motivates the CuO nanomaterials and generates electrons over their surface [71,72,73]. These electrons are represented in J_ph_ values and the H_2_ generation reaction rate [74,75].

## 4. Conclusions

This work provides promising results in support of H_2_ production from sewage water using a CuO NP photoelectrode. All the analyses were carried out for confirming the chemical structure, morphology, and optical properties of the prepared CuO NP. From the SEM, the prepared materials have nanoporous features that look like small islands with diameters of about 300–400 nm, with each island composed of a package of small nanoporous particles. XRD confirmed the monoclinic CuO crystalline phase with crystallite size of 89 nm. The obtained optical band gap value for CuO NP was 1.39 eV. The sewage water was used as a source of H_2_ gas produced by the Cu/CuO photoelectrode. The J_ph_ value was changed from 2.17 to 4.7 mA·cm^−2^ upon increasing the light power density from 50 to 100 mW·cm^−2^, respectively. The IPCE was changed under the effect of monochromatic light, in which the optimum IPCE value was 14.6% at 390 nm. Moreover, the effect of on and off chopped current was studied that confirms the motivation of the photocatalyst under light incidence. The sewage water splitting thermodynamics were studied, in which the enthalpy (ΔH*) and entropy (ΔS*) values for the Cu/CuO electrode were 9.519 kJ·mol^−1^ and 180.4 JK^−1^·mol^−1^, respectively. A mechanism was proposed to explain the relationship between the incidence light and the J_ph_ values of the H_2_ generation rate.

## Figures and Tables

**Table 1:** The sewerage water chemical composition used as the electrolyte for H_2_ production.

Material or Element	Concentration (mg/L)
Phenols	0.015
F^−^	1.0
Al^3+^	3.0
NH_3_	5.0
Hg^2+^	0.005
Pb^2+^	0.5
Cd^3+^	0.05
As^3+^	0.05
Cr^3+^	1.0
Cu^2+^	1.5
Ni^3+^	0.1
Fe^3+^	1.5
Mn^2+^	1.0
Zn^2+^	5.0
Ag^+^	0.1
Ba^3+^	2.0
Co^2+^	2.0
Other cations	0.1
Pesticides	0.2
CN^−1^	0.1
Industrial washing	0.5
Coli groups	4000/100 cm^3^

**Table 2:** Comparison between the present study and the previous reported literature; electrolyte used, J_ph_, and IPCE values.

Photoelectrode	Electrolyte	J_ph_ (mA/cm^2^)	Applied Voltage (V)	IPCE% (390 nm)	Light Source
g-C_3_N4-CuO [23]	NaOH	0.01	1.6	-	300 W xenon lamp
CuO-C/TiO_2_ [76]	glycerol	0.001	−0.5	-	300 W xenon lamp
CuO nanowire [77]	Na_2_SO_4_	1.5	−0.5	-	simulated AM1.5 illumination
CuO nanostructure [78]	KOH	1	−1.2	-	White light
CuO thin films [79]	Na_2_SO_4_	2.5	0	3.1	Solar simulator 1.5 global (AM 1.5G)
CuO nanocrystals [80]	Na_2_SO_4_	1.1	−0.5	8.7	Xenon lamp light
TiO_2_/CdS/PbS [81]	Na_2_S/Na_2_S_2_O_3_	2	0.2	4	AM 1.5G illumination
GaN [82]	HBr	0.6	+1	8	Sunlight
ZnO/TiO_2_/FeOOH [83]	Na_2_S_2_O_3_	1.59	0.8	-	A 150 W xenon lamp
SnO_2_/TiO_2_ [84]	Na_2_S_2_O_3_	0.4	0.6	-	1 Sun (100 mW cm^−2^)
Au/PbS/Ro-GO/PANI [85]	Na_2_S_2_O_3_	1.1	+1	10	400 W xenon lamp
TiN-TiO_2_ [86]	NaOH	3.0 × 10^−4^	0.2	0.03	Solar simulator (150 mW cm^−2^)
BiFeO_3_ [29]	NaOH	0.1	1.6	0.21	1 sun (AM 1.5G solar spectrum)
ITO/VO2 [50]	Na_2_S_2_O_3_	1.5	+1	4	400 W metal halid
PrFeO [49]	Na_2_SO_4_	0.130	−0.6	-	Simulated sunlight
Poly(3-aminobenzoic acid) frame [53]	H_2_SO_4_	1.2	1.6	-	150 W xenon lamp
Cu/CuO (Present work)	Sewage water	4.7	+1	14.6	Simulated sunlight

## Data Availability

The data that support the findings of this study are available from the corresponding author upon reasonable request.
